# Genetically confirmed first records of an egg and a juvenile roundscale spearfish, *Tetrapturus georgii*

**DOI:** 10.1111/jfb.16043

**Published:** 2025-01-02

**Authors:** Marko Freese, Tina Blancke, Lasse Marohn, Jan‐Dag Pohlmann, Josefin Sundin, Klaus Wysujack, Reinhold Hanel

**Affiliations:** ^1^ Federal Research Institute for Rural Areas, Forestry and Fisheries Thünen Institute of Fisheries Ecology Bremerhaven Germany; ^2^ Department of Aquatic Resources Swedish University of Agricultural Sciences Drottningholm Sweden

**Keywords:** barcode, ichthyoplankton, Isaacs‐Kidd Midwater Trawl, Istiophoridae, marlin, Sargasso Sea, *Tetrapturus georgii*

## Abstract

The roundscale spearfish (*Tetrapturus georgii*) is a poorly studied species with limited information available on its biology, ecology, and population status. Although the adult life stage of the species is morphologically distinguishable from closely related species such as the overexploited white marlin (*Kajikia albida*), misidentification is common, adding to the uncertainties connected with population assessments of these pelagic highly migratory species. Although genetic studies have recently confirmed its distinction from congeneric species, much of the reproductive biology and population dynamics of *T. georgii* remain unknown, underscoring the need for further research to guide conservation and management strategies. This study reports the first documented records of an egg and a juvenile *T. georgii*. Here, we provide photographs, morphological descriptions, and collection site data for genetically confirmed egg and juvenile specimens obtained during two multipurpose research surveys in the Sargasso Sea Subtropical Convergence Zone. These findings contribute new insights into the reproductive biology, early life stages, and ecology of this elusive species.

## 1 INTRODUCTION

Understanding the reproductive biology and early life stages of fish is crucial for both basic and applied research, as well as for effective management, particularly for exploited species. Detailed knowledge of the temporal and spatial distribution of species at different life‐history stages, along with the biological and physical factors that influence recruitment, can provide critical insights into the species' ecological strategies, spawning behavior, and stock sizes. Such information is essential for the development and implementation of successful conservation and management strategies.

Marlins, belonging to the family Istiophoridae, comprise 9 to 11 known species of highly migratory euteleosts that are widely distributed across tropical‐to‐temperate seas (Froese & Pauly, 2024; Graves & McDowell, 2015). The discrepancy between referencing 9 and 11 species arises from ongoing discussions regarding the divergence between Atlantic and Indo‐Pacific populations of blue marlin (*Makaira nigricans*/*Makaira mazara*) and sailfish (*Istiophorus albicans/Istiophorus platypterus*) (Bernard et al., 2013; Collette et al., 2006; Froese & Pauly, 2024; Graves & McDowell, 2015; Nakamura, 1985). Istiophorids are large pelagic predators typically found in deeper, offshore, and high‐seas environments (Shivji et al., 2006). Several species within this group hold significant value in commercial and recreational fisheries (Beerkircher et al., 2009; Schmidt et al., 2015). One of the lesser‐studied species within this family is the roundscale spearfish, *Tetrapturus georgii*. The lack of existing studies can, in part, be due to the fact that this species has long been misidentified as the white marlin, *Kajikia albida* (Beerkircher et al., 2009; Beerkircher & Serafy, 2011; Shivji et al., 2006). *T. georgii* was first described in 1840 based on the morphological characteristics of a single specimen (Lowe, 1840), with a more comprehensive description following in 1961 based on four specimens (Robins, 1974). However, the species' existence remained uncertain for decades until Shivji et al. (2006) conducted further morphological and genetic analyses on 16 specimens of *T. georgii*, extending its known distribution and confirming its validity as a distinct species. Updated morphological descriptions and key identification characteristics of adult *T. georgii* for the western North Atlantic were later published by Beerkircher et al. (2008). Bernard et al. (2013) then utilized genetically confirmed data to further describe the distribution range of *T. georgii* and highlighted the management complexities, as the geographic distribution overlaps with both the white marlin and the longbill spearfish (*Tetrapturus pfluegeri*), which increases the risks of species misidentification between *T. georgii* and *K. albida* in catch records. Despite these advancements, nearly 20 years have passed with minimal additional knowledge gained regarding the species' stock status, population trends, movements, and reproductive biology. Consequently, *T. georgii* remains classified as “data deficient” in the IUCN Red List of Threatened Species assessment (Collette et al., 2023). Similar to other marlin species, it is expected that the early life history of the roundscale spearfish includes a pelagic egg phase followed by a planktonic larval stage. A few descriptions of eggs and early larvae of other Istiophoridae are available in scientific literature (e.g., Luthy et al., 2005; Rodrigues et al., 2017; Ueyanagi & Wares, 1975), and some illustrated larvae can be found in early life‐stage identification keys (Richards, 2005). Rodrigues et al. (2017) were likely the first to use molecular biological methods to identify billfish larvae and eggs (Istiophoridae and Xiphiidae), and reported the first molecular confirmation of sailfish (*I. platypterus*) and white marlin (*K. albida*) larvae, as well as swordfish (*Xiphias gladius*) eggs along the Brazilian coast. Although *T. georgii* was referenced in their publication as a species of interest for future research, almost no information about the early life history of *T. georgii* can be found to date. For instance, there are no descriptions of eggs, larvae, or juveniles in comprehensive identification guides for early life stages of Atlantic fishes (e.g., Richards, 2005); no data on sexual dimorphism, size at first maturity, spawning time/season, or spawning details (Collette, 2010; Collette et al., 2023); and no valid information on maturity, reproduction, spawning, eggs, fecundity, or larvae in the online fish database FishBase (Froese & Pauly, 2024). Some information about length and age at first maturity, spawning duration, and batch fecundity (number of ova) is presented in a table found in Salcedo‐Bojorquez and Arreguin‐Sanchez (2011); however, the referenced sources (Arocha & Ortiz, 2006; Nakamura, 1985) do not contain this information.

The aim of this study was to, for the first time, describe the egg and juvenile life stages of *T. georgii*. The description is based on one egg and one juvenile specimen collected during two multipurpose research surveys conducted in the Sargasso Sea Subtropical Convergence Zone, south of Bermuda.

## 2 MATERIALS AND METHODS

### 2.1 Sargasso Sea research survey

**FIGURE 1:**
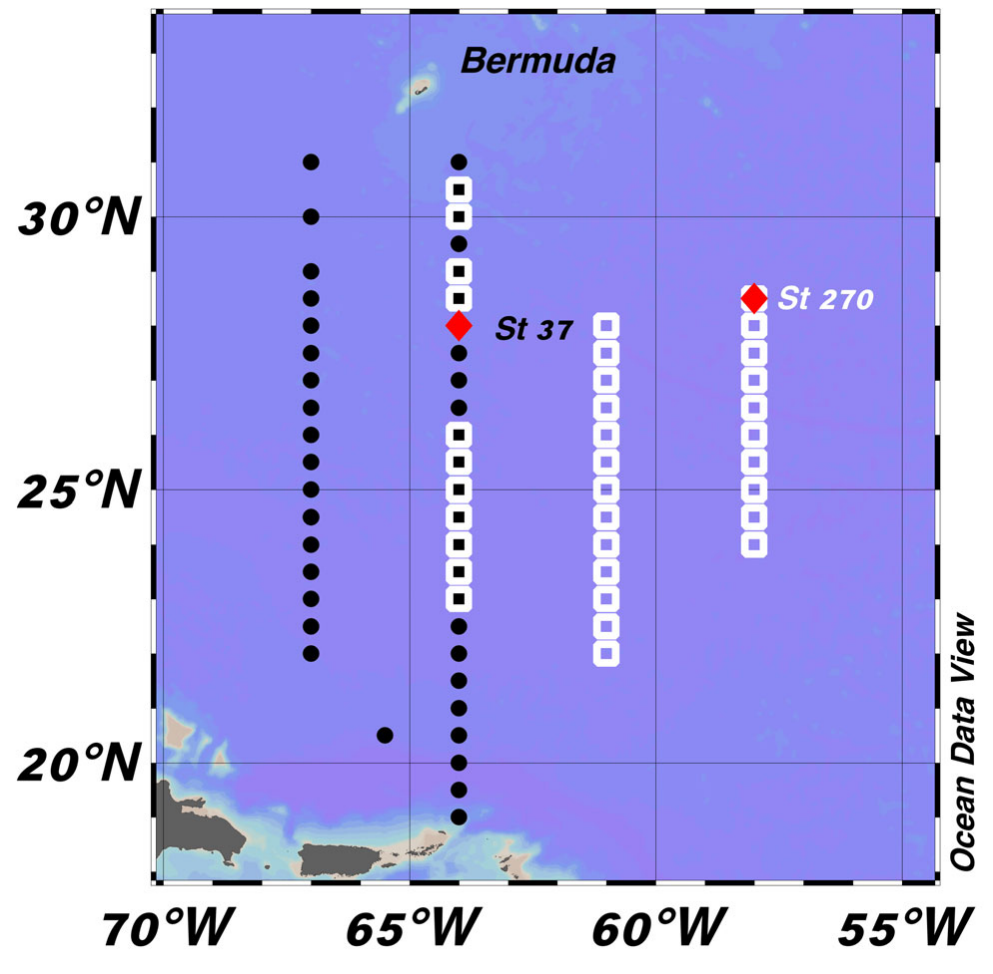
Station grids of two multipurpose research surveys conducted between March and April in 2017 (cruise number WH404, white squares) and 2023 (cruise number WH465, black dots) in the central Sargasso Sea. Locations (stations) where the roundscale spearfish, *Tetrapturus georgii*, egg (St 37 in 2023, WH465), and juvenile (St 270 in 2017, WH404) were collected are labeled and indicated by red diamonds.

The egg and juvenile samples of the roundscale spearfish were collected during two separate multipurpose research surveys conducted between March and April in 2017 and 2023 aboard the German fishery research vessel *Walther Herwig III* (cruise numbers WH‐404 and WH‐465). The primary objective of the triennial survey was to investigate the distribution, abundance, and ecological aspects of the early life‐history stages of the European eel (*Anguilla anguilla*) and American eel (*Anguilla rostrata*). The sampling design and specific methods of the recurring survey have been described in previous publications focusing on different species (Hellenbrecht et al., 2019; Miller et al., 2019; Sundin et al., 2023) and are, therefore, briefly described here. During the 2017 and 2023 cruises, a total of 35 and 45 stations, respectively, were sampled along two north–south transects. Sampling took place between 22° to 30°30′N and 58° to 64° W in 2017 (WH404) and between 19° to 31° N and 64° to 67° W in 2023 (WH465) (Figure 1). Sampling stations were spaced 0.5° to 1° apart along the latitudinal transects. Water depth in the sampled area ranged from approximately 5000 to 7000 m.

### 2.2 Ichthyoplankton sampling

**FIGURE 2:**
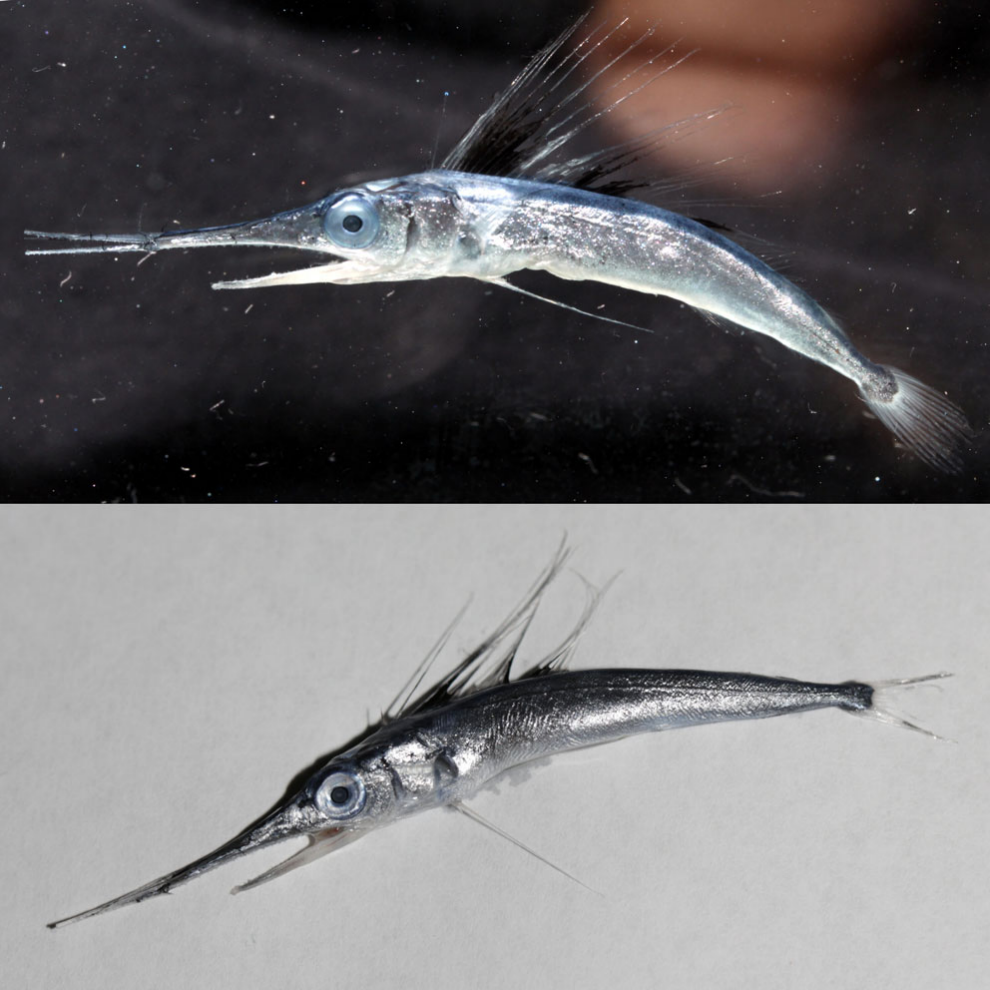
Photographs of a genetically confirmed, juvenile roundscale spearfish *Tetrapturus georgii* (total length: 64.0 mm, lower jaw fork length: 46.3 mm, and eye diameter: 3.7 mm), collected in the Sargasso Sea using an Isaacs‐Kidd Midwater Trawl during multipurpose research survey WH404 between March and April 2017.

**FIGURE 3:**
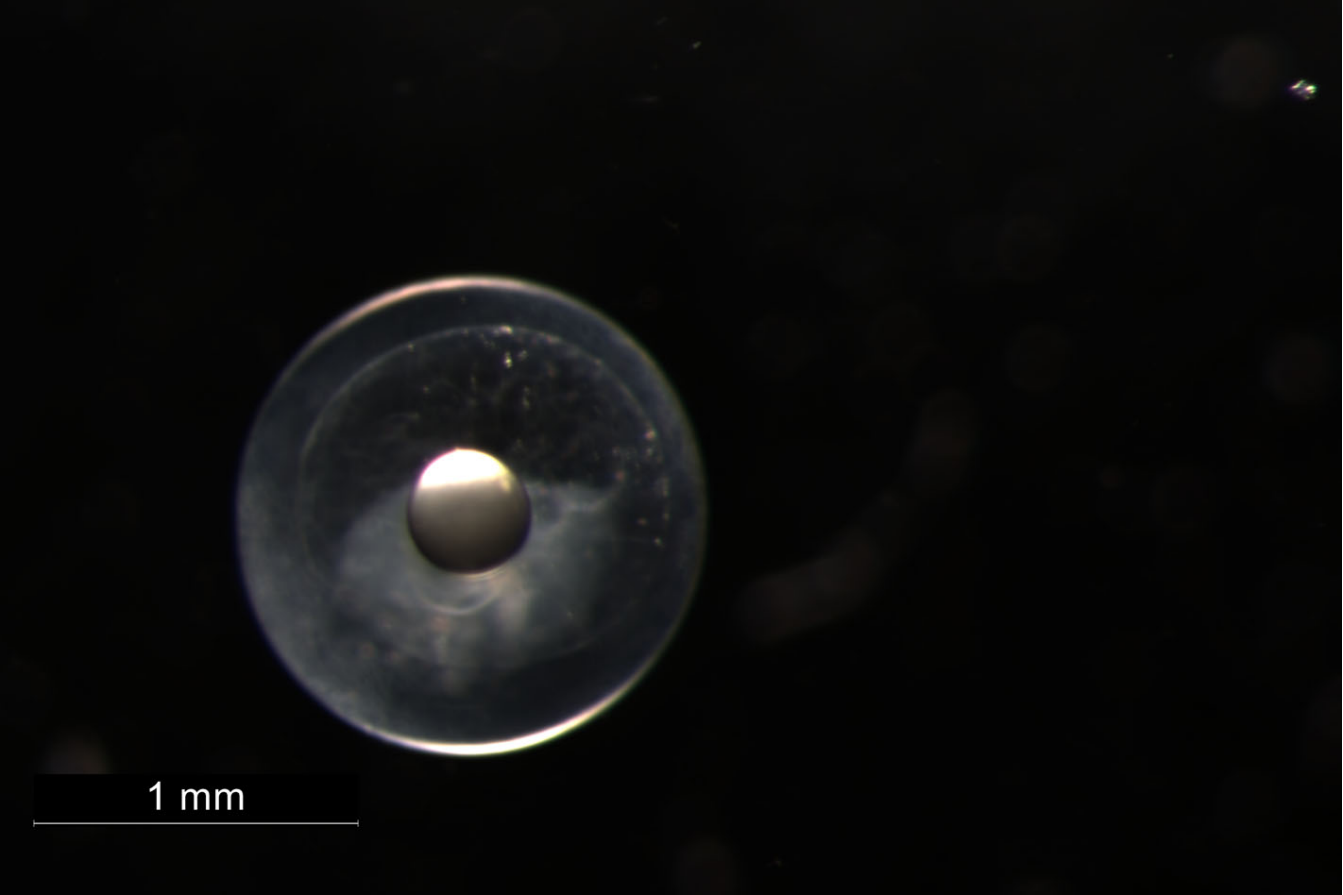
Photograph of a genetically confirmed egg (egg diameter: 1.451 mm, conceptus diameter: 1.149 mm, and oil droplet diameter: 0.379 mm) of the roundscale spearfish, *Tetrapturus georgii*, collected in the Sargasso Sea using an Isaacs‐Kidd Midwater Trawl during multipurpose research survey WH 465 conducted between March and April 2023.

Sampling was conducted using an Isaacs‐Kidd Midwater Trawl (IKMT) with a mesh‐size of 500 μm, a mouth opening of 6.2 m^2^, and a total length of 10 m (Hydro‐Bios Apparatebau GmbH). The IKMT was deployed using double‐oblique tows between the surface and a maximum depth of 300 m during night and day. Plankton samples were manually sorted onboard immediately upon collection. The juvenile specimen (Figure 2), identified as *T. georgii*, was collected in 2017. It was identified to the family level onboard based on a region‐specific identification guide (Richards, 2005), photographed using a Canon EOS 5D Mark III camera with a 100‐mm F 2.8‐L macro lens; and its total length was measured on a ruler before it was fixed in ethanol (99%) and stored at room temperature. The egg (Figure 3) was collected among other unidentified eggs in 2023. After being sorted from the plankton sample onboard, the egg was kept at +4°C until detailed observation and photographing with a Leica M125C stereo microscope fitted with a DMC 5400 camera (Leica Mikrosysteme Vertrieb GmbH, Wetzlar, Germany). The diameter of the egg was digitally measured using the analysis tool provided by Leica Application Suite Software version 4.13. Afterward, it was transferred into a reaction tube containing 100‐μL of 5% Chelex100 solution (using clean tweezers) and kept at +4°C until DNA extraction. The DNA sample was frozen at −20°C until further genetic processing at the Thünen Institute of Fisheries Ecology, Bremerhaven, Germany.

### 2.3 Genetic species identification

Genetic species identification was conducted by DNA barcoding and Basic Local Alignment Search Tool (BLAST) analysis at the Thünen Institute of Fisheries Ecology, Bremerhaven, Germany. In short, genomic template DNA from both samples was extracted by incubating the tissue pieces in 100 μL of 5%–10% Chelex100 at 95°C for 20 min according to standard protocols (Walsh et al., 1991). The used amounts of tissue for Chelex extraction were the entire fresh single egg and approximately 1 mm^3^ of eye tissue from the ethanol‐preserved juvenile, each crushed by micro‐pestle within the Chelex tube. As a standard procedure for unidentified fish tissue, both DNA samples were amplified using PCR for the DNA barcoding marker genes cytochrome c oxidase I (COI) and 16S ribosomal RNA (rRNA) with primers given in Table 1, as described in Marohn et al. (2021) for COI and in Hellenbrecht et al. (2019) for 16S. For the genetic analyses of unidentified eggs found in our surveys, 16S is used as a first barcoding marker gene as a standard method. The sequencing of the PCR products was carried out by a service laboratory (StarSEQ GmbH, Mainz, Germany), and forward and reverse DNA sequences were checked and trimmed before generating consensus COI sequences for each sample. For species identification, the sequences of both samples were analysed by nucleotide BLAST against National Center for Biotechnology Information (NCBI) database (Altschul et al., 1990) and aligned for each barcoding gene with BioEdit Sequence Alignment Editor version 7.2.5 (Hall, 1999). Genetic distances were calculated pair‐wise and compared to istiophorid reference sequences (Table 2) obtained from NCBI's GenBank using the DNADist function as implemented in BioEdit. For both, pair‐wise and multiple alignments, all sequences were trimmed to the same length set by the shortest sequence prior to identity calculation.

**TABLE 1:** Barcoding gene primer information.

Primer	Sequence (5′‐3′)	Barcoding gene	Origin
VF2_t1‐M13	TGTAAAACGACGGCCAGTCAACCAACCACAAAGACATTGGCAC	COI	Ivanova et al., 2007
FishF2_t1‐M13	TGTAAAACGACGGCCAGTCGACTAATCATAAAGATATCGGCAC		
FishR2_t1‐M13	CAGGAAACAGCTATGACACTTCAGGGTGACCGAAGAATCAGAA		
FR1d_t1‐M13	CAGGAAACAGCTATGACACCTCAGGGTGTCCGAARAAYCARAA		
16L29	YGCCTGTTTATCAAAAACAT	16S	Schubart et al., 2001
H3059	CCGGTCTGAACTCAGATCACGT		Palumbi, 1996

**TABLE 2:** Istiophorid reference sequences from GenBank (National Center for Biotechnology Information [NCBI]) used for multiple sequence alignments.

Accession	Description	Gene
HM071033.1	*Tetrapturus georgii* 16S ribosomal RNA gene, partial sequence; mitochondrial	16S
KU315123.1	*T. georgii* isolate D voucher TEGE0001 mitochondrion, complete genome	16S/COI
HQ024848.1	*T. georgii* voucher 07‐GHRI‐0467 cytochrome oxidase subunit I (COI) gene, partial cds; mitochondrial	COI
HM071009.1	*T. georgii* COI gene, complete cds; mitochondrial	COI
HM909850.1	*T. georgii* voucher *T. georgei*‐OC‐149 COI gene, partial cds; mitochondrial	COI
MT455453.1	*T. georgii* voucher USNM:FISH:451153 COI gene, partial cds; mitochondrial	COI
MT455942.1	*T. georgii* voucher USNM:FISH:451157 COI gene, partial cds; mitochondrial	COI
NC_030008.1	*Tetrapturus belone* isolate C voucher TEBE0001 mitochondrion, complete genome	16S/COI
NC_030007.1	*Tetrapturus pfluegeri* isolate B voucher TEPF0001 mitochondrion, complete genome	16S/COI
AB470303.1	*Tetrapturus angustirostris* mitochondrial DNA, complete genome	16S/COI
NC_030010.1	*Kajikia albida* isolate G voucher KAAL0001 mitochondrion, complete genome	16S/COI
KU315126.1	*Kajikia audax* isolate F voucher KAAU0001 mitochondrion, complete genome	16S/COI
HQ592244.1	*Makaira nigricans* voucher 09061C16S 16S ribosomal RNA gene, partial sequence; mitochondrial	16S
OQ386437.1	*M. nigricans* voucher PIL‐191 COI gene, partial cds; mitochondrial	COI
KJ510417.1	*Istiompax indica* voucher ISIN1005 mitochondrion, complete genome	16S/COI
AB470306.1	*Istiophorus platypterus* mitochondrial DNA, complete genome	16S
OP404150.1	*I. platypterus* isolate SFA59 mitochondrion, complete genome	16S/COI
GQ202123.2	*I. platypterus* COI gene, partial cds; mitochondrial	COI

## 3 RESULTS AND DISCUSSION

**FIGURE 4:**
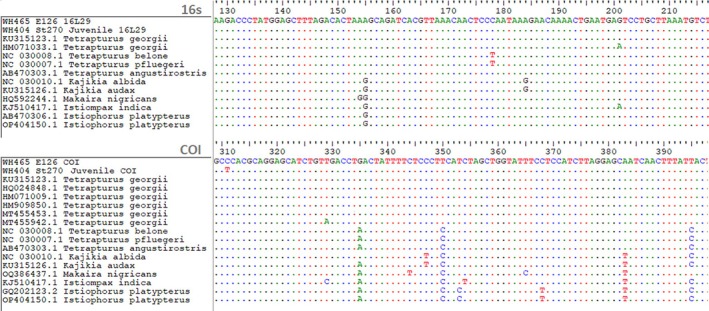
Image details of the 16S and cytochrome c oxidase I (COI) sequence alignment (BioEdit Sequence Alignment Editor), with the first two rows showing the survey samples from this study followed by istiophorid sequences from GenBank. Identical bases compared to the reference sequence on top are indicated by points.

This study provides the first genetically confirmed records of egg and juvenile *T. georgii*, revealing new insights into the species' possible spawning and nursery areas. Genetic confirmation of both the egg and the juvenile samples was clear: BLAST results for the egg showed 100% identity for *T. georgii* sequence entries in both barcoding genes, generating 15 hits for COI and 1 for 16S. The juvenile specimen also showed 100% identity for one of the *T. georgii* entries in 16S and 99.84% for the first seven hits in COI. For 16S, analyses resulted in a 533‐bp‐long sequence for the egg and 503 bp for the juvenile sailfish, showing absolute conformity for the overlapping region in a pair‐wise alignment. For COI, consensus sequences were generated with a length of 552 bp for the egg and 619 bp for the sampled juvenile, resulting in 0.9963768 identities with two different nucleotides in the sample alignment. Aligned to other istiophorid sequences obtained from GenBank (Figure 4), the two samples were consequently identified as *T. georgii*, as they were clearly distinguished from other related species, in particular regarding COI. Genetic distances between the egg, the juvenile, and istiophorid sequences from GenBank for both barcoding gene markers are presented in Tables 3 and 4. For 16S, interspecific sequence variations were too small for an unambiguous discrimination between the istiophorid species, as both samples showed equal genetic distance values (0.0020) to reference sequences of *T. georgii* (account number HM071033.1) and *I. platypterus* (account number AB470306.1). Due to scarcity of *T. georgii* entries for 16S in GenBank (*n* = 3), with two of them even representing the same voucher specimen, COI with more than 50 sequence entries was chosen as the alternative barcoding marker. With a mean p‐distance value of 0.0021 for the egg and 0.0039 for the sampled juvenile, the genetic distances in COI (Table 4) for both samples, when pair‐wise compared to GenBank's *T. georgii* sequences, clearly differed from those compared to all other istiophorid sequences (egg = 0.0341; juvenile = 0.0358), despite relatively high intraspecific variations within *T. georgii* sequences ranging from 0.0000 to 0.0073.

**TABLE 3:** Pair‐wise genetic distances (16S) of egg and juvenile *Tetrapturus georgii* with 11 istiophorid sequences obtained from National Center for Biotechnology Information (NCBI's) GenBank as calculated by BioEdit DNADist.

Survey or NCBI sample sequence	NCBI account number	WH465 egg (E126)	WH404 juvenile (St.270)
WH465 egg (St 37)	‐	‐	0.0000
WH404 juvenile (St 270)	‐	0.0000	‐
Roundscale spearfish (*Tetrapturus georgii*)	KU315123.1	0.0000	0.0000
Roundscale spearfish *(Tetrapturus georgii*)	HM071033.1	0.0020	0.0020
Mediterranean spearfish (*Tetrapturus belone*)	NC_030008.1	0.0040	0.0040
Longbill spearfish (*Tetrapturus pfluegeri*)	NC_030007.1	0.0040	0.0040
Shortbill spearfish (*Tetrapturus angustirostris*)	AB470303.1	0.0040	0.0040
White marlin (*Kajikia albida*)	NC_030010.1	0.0040	0.0040
Striped marlin (*Kajikia audax*)	KU315126.1	0.0040	0.0040
Blue marlin (*Makaira nigricans*)	HQ592244.1	0.0080	0.0080
Black marlin (*Istiompax indica*)	KJ510417.1	0.0060	0.0060
Indo‐Pacific sailfish (*Istiophorus platypterus*)	AB470306.1	0.0020	0.0020
Indo‐Pacific sailfish (*I. platypterus*)	OP404150.1	0.0020	0.0020

**TABLE 4:** Pair‐wise genetic distances (COI) of the two survey samples with 15 istiophorid sequences obtained from National Center for Biotechnology Information (NCBI's) GenBank as calculated by BioEdit DNADist.

Survey or NCBI sample sequence	NCBI account number	WH465 egg (E126)		WH404 juvenile (St.270)	
WH465 egg (St 37)	‐	‐	‐	0.0036	‐
WH404 juvenile (St 270)	‐	0.0036	‐	‐	‐
Roundscale spearfish (*Tetrapturus georgii*)	KU315123.1	0.0000	Ø 0.0021	0.0036	Ø 0.0039
Roundscale spearfish (*T. georgii*)	HQ024848.1	0.0000		0.0036	
Roundscale spearfish (*T. georgii*)	HM071009.1	0.0036		0.0073	
Roundscale spearfish (*T. georgii*)	HM909850.1	0.0036		0.0036	
Roundscale spearfish (*T. georgii*)	MT455453.1	0.0018		0.0018	
Roundscale spearfish (*T. georgii*)	MT455942.1	0.0036		0.0036	
Mediterranean spearfish (*Tetrapturus belone*)	NC_030008.1	0.0202	Ø 0.0341	0.0202	Ø 0.0358
Longbill spearfish (*Tetrapturus pfluegeri*)	NC_030007.1	0.0202		0.0202	
Shortbill spearfish (*Tetrapturus angustirostris*)	AB470303.1	0.0221		0.0221	
White marlin (*Kajikia albida*)	NC_030010.1	0.0315		0.0315	
Striped marlin (*Kajikia audax*)	KU315126.1	0.0334		0.0334	
Blue marlin (*Makaira nigricans*)	OQ386437.1	0.0391		0.0429	
Black marlin (*Istiompax indica*)	KJ510417.1	0.0428		0.0466	
Indo‐Pacific sailfish (*Istiophorus platypterus*)	GQ202123.2	0.0486		0.0525	
Indo‐Pacific sailfish (*I. platypterus*)	OP404150.1	0.0486		0.0525	

The juvenile specimen and the egg were collected in the Sargasso Sea Subtropical Convergence Zone at 58°00′W 28°30′N (juvenile, station 270, WH404 in 2017) and 64°00′W 28°00′N (egg, station 37, WH465 in 2023), respectively. The locations fit well with the known latitudinal distribution of *T. georgii*, which ranges from 37°41′N off the coast of New Jersey, USA, to 28°52′S, off the coast of southern Brazil, according to a study based on genetically verified individuals (Bernard et al., 2013). However, Beerkircher et al. (2008) report that the northern latitudinal range of the species extends to north of 40° for identifications based on laboratory‐examined individuals and to north of 45° for identifications based on scale samples and ventral/anal‐fin location. The known longitudinal distribution ranges from just off the coast of Florida (USA), the Caribbean Sea, the western and eastern Atlantic near Madeira, but apparently also into the Mediterranean, where a single individual has been reported from near Sicily, Italy, and another from the Strait of Gibraltar (Bernard et al., 2013; Robins, 1974). Both samples presented here, taken at latitudes of 28°00′N and 28°30′N, have their origin in the Sargasso Subtropical Convergence Zone, an area known to serve as a spawning area for a variety of species (Ayala et al., 2016; Hellenbrecht et al., 2019; Miller et al., 2019). Regarding seasonality, the collection of a juvenile on April 1 and an egg on April 4 correspond well with the estimated spawning season of the closely related white marlin (*K. albidus*), which are thought to spawn from March to June, and the longbill spearfish (*T. pfluegeri*), which are thought to spawn from November to May (de Sylva & Breder, 1997). Marlins are fast‐growing species. In a study by Prince et al. (1991), a juvenile blue marlin (*M. nigricans*) with a lower jaw fork length (LJFL) of 23.0 cm was estimated to be 40 days old (with a range of 31–60 days) based on daily otolith ring counts. Given this information, the individual collected on April 1, measuring 4.63 cm LJFL, is likely also just a few weeks old (Figure 2). This would also align well with the known spawning seasons of the related species. Photographs of the egg (diameter: 1.451 mm) taken at the time of collection show an oil droplet with a diameter of 0.379 mm and, based on the clearly recognizable perivitelline space, a fertilized zygote (conceptus diameter: 1.149 mm) can be identified (Figure 3). Because no embryo or signs of advanced cell differentiation are recognizable in our sample, an early stage of fertilization and, thus, a temporal as well as spatial proximity of the sampling to the spawning site and time can be assumed. Istiophorids are thought to be batch‐spawners that spawn more than once during their spawning season, which is shown by simultaneous appearance of multiple stages of ovulated eggs, ova, oocytes, and oogonia in the ovaries of several species (de and Breder, 1997). Ueyanagi et al. (1970a; 1970b) reported maturity data from white marlin from different areas, which support the hypothesis that the species could be ready to spawn from October to March in the southwestern Atlantic off the coast of Brazil. It is known, though, that spawning activity of *K. albidus* has also been reported from multiple areas, including oceanic waters near Florida, Puerto Rico, and the Caribbean, from April to June (Arocha et al., 2005; Arocha & Ortiz, 2006; Baglin Jr., 1979). Even though the finding of one egg and one early juvenile provides evidence for spawning activity in the research area presented here, it remains entirely unclear if this is just one of multiple possible spawning locations and timings for *T. georgii*. To obtain a better geographical and temporal coverage of the occurrence of early life stages of billfishes in the Sargasso Sea, an intensified ichthyoplankton sampling conducted at different seasons would be required. Such research would provide more detailed information on the spawning behavior and timing of *T. georgii*, helping to clarify the ecological role of the Sargasso Sea as a potential spawning and nursery area for this species.

## AUTHOR CONTRIBUTIONS

Conceptualization: Marko Freese and Tina Blancke. Methodology: Marko Freese, Tina Blancke, Lasse Marohn, Jan‐Dag Pohlmann, Klaus Wysujack, Josefin Sundin, and Reinhold Hanel. Formal analysis: Marko Freese and Tina Blancke. Resources: Marko Freese, Lasse Marohn, Reinhold Hanel, and Josefin Sundin. Data curation: Marko Freese, Tina Blancke, and Lasse Marohn. Writing—original draft: Marko Freese. Writing, review, and editing: Marko Freese, Tina Blancke, Lasse Marohn, Jan‐Dag Pohlmann, Klaus Wysujack, Josefin Sundin, and Reinhold Hanel. Visualization: Marko Freese and Tina Blancke.

## FUNDING INFORMATION

The R.V. Walther Herwig III survey was funded by the German Federal Ministry of Food and Agriculture. Additional funding was obtained from the Siemiatkowski Foundation (to Josefin Sundin). Jan‐Dag Pohlmann, Lasse Marohn and Marko Freese are co‐funded by the European Commission's Data Collection Framework (DCF).

## CONFLICT OF INTEREST STATEMENT

The authors have no conflict of interest to declare.

## DECLARATION OF GENERATIVE ARTIFICIAL INTELLIGENCE AND ARTIFICIAL INTELLIGENCE–ASSISTED TECHNOLOGIES IN THE WRITING PROCESS

During the preparation of the first draft of this work, Marko Freese made use of the artificial intelligence (AI)‐assisted translation software DeepL (http://www.deepl.com) and ChatGPT (https://chatgpt.com) for linguistic and stylistic proofreading of the manuscript. After using these tools, the authors reviewed and edited the content as needed and take full responsibility for the content of the publication.
